# Mettl3-mediated m^6^A modification plays a role in lipid metabolism disorders and progressive liver damage in mice by regulating lipid metabolism-related gene expression

**DOI:** 10.18632/aging.204810

**Published:** 2023-06-16

**Authors:** Guanqi Dai, Shihao Huang, Yonglong Li, Xueyi Tu, Jiawei Xia, Zhihao Zhou, Wanyi Chen, Ao Zhang, Jintao Lin, Yingchun Li, Danhua He, Taoyan Lin, Jinge Cong, Ye Lei, Liuxin Han, Zhenxia Yao, Weiwei Liu, Ying Zhou, Qiwen Li, Jing Li, Yuqin Zhang, Aibing Wu, Dong Xiao, Wanshan Wang, Wentao Zhao, Junshuang Jia, Xiaolin Lin

**Affiliations:** 1 Cancer Research Institute, Experimental Education/Administration Center, School of Basic Medical Sciences, Southern Medical University, Guangzhou 510515, China; 2 Laboratory Animal Management Center, Southern Medical University, Guangzhou 510515, China; 3 The Third People’s Hospital of Kunming (The Sixth Affiliated Hospital of Dali University), Kunming 650041, China; 4 Department of Pharmacy, Nanfang Hospital, Southern Medical University, Guangzhou 510515, China; 5 Radiotherapy Center, The First People's Hospital of Chenzhou, Xiangnan University, Chenzhou 423000, China; 6 Department of Radiation Oncology, Nanfang Hospital, Southern Medical University, Guangzhou 510515, China; 7 Department of Oncology, The Central People's Hospital of Zhanjiang, Zhanjiang 524000, China; 8 Department of Gastrointestinal Oncology, The Third Affiliated Hospital of Kunming Medical University (Yunnan Cancer Hospital, Yunnan Cancer Center), Kunming 650118, China; 9 Cancer Center, Integrated Hospital of Traditional Chinese Medicine, Southern Medical University, Guangzhou 510315, China

**Keywords:** NAFLD, m^6^A modification, epigenetics, lipid metabolism, Mettl3

## Abstract

Aims: N6-methyladenosine (m^6^A), the most abundant and conserved epigenetic modification of mRNA, participates in various physiological and pathological processes. However, the roles of m^6^A modification in liver lipid metabolism have yet to be understood entirely. We aimed to investigate the roles of the m^6^A “writer” protein methyltransferase-like 3 (Mettl3) in liver lipid metabolism and the underlying mechanisms.

Main Methods: We assessed the expression of Mettl3 in liver tissues of diabetes (db/db) mice, obese (ob/ob) mice, high saturated fat-, cholesterol-, and fructose-induced non-alcoholic fatty liver disease (NAFLD) mice, and alcohol abuse and alcoholism (NIAAA) mice by quantitative reverse-transcriptase PCR (qRT-PCR). Hepatocyte-specific Mettl3 knockout mice were used to evaluate the effects of Mettl3 deficiency in mouse liver. The molecular mechanisms underlying the roles of Mettl3 deletion in liver lipid metabolism were explored by multi-omics joint analysis of public data from the Gene Expression Omnibus database and further validated by qRT-PCR and Western blot.

Key Findings: Significantly decreased Mettl3 expression was associated with NAFLD progression. Hepatocyte-specific knockout of Mettl3 resulted in significant lipid accumulation in the liver, increased serum total cholesterol levels, and progressive liver damage in mice. Mechanistically, loss of Mettl3 significantly downregulated the expression levels of multiple m^6^A-modified mRNAs related to lipid metabolism, including Adh7, Cpt1a, and Cyp7a1, further promoting lipid metabolism disorders and liver injury in mice.

Significance: In summary, our findings demonstrate that the expression alteration of genes related to lipid metabolism by Mettl3-mediated m^6^A modification contributes to the development of NAFLD.

## INTRODUCTION

The liver is an essential organ with complex functions, including metabolism, drug detoxification, and hormone secretion [1]. It plays a central role in lipid homeostasis, including the synthesis of new fatty acids, uptake of circulating fatty acids, biosynthesis of triglycerides, and fatty acid oxidation [2], and disruption of one or more of these pathways may cause lipid metabolism disorder, which is a major contributing factor to cardiovascular diseases and nonalcoholic fatty liver disease (NAFLD) [3]. Recently, increasing evidence has shown that hepatic cholesterol accumulation, characterized by elevated cholesterol synthesis, uptake from circulating lipoproteins, and reduced cholesterol excretion, contributes to the pathogenesis of NAFLD [4–6]. Thus, identifying the key regulators of lipid metabolism may provide effective treatment measures for NAFLD.

The critical role of epigenetic modifications in metabolic diseases has been widely reported [7, 8]. Among these medications, the N6-methyladenosine (m^6^A) modification is the most prevalent internal modification of mRNAs. It is catalyzed by a series of m^6^A “writers,” such as methyltransferase-like 3 (Mettl3) [9], Mettl14 [10], Mettl16 [11], and Wilms tumor 1-associated protein (WTAP) [12]. It can be removed by RNA demethylases, including fat mass and obesity-associated protein (FTO) [13] and alkylation repair homolog protein 5 (ALKBH5) [14]. This modification can be recognized by a set of YHT family domain-containing “reader” proteins (Ythdf1/2/3) [15, 16] or insulin-like growth factor-2 mRNA-binding proteins (Igfbp1/2/3) [17] and regulates gene expression by affecting multiple aspects of mRNA metabolism, such as RNA splicing, RNA stability, and mRNA translation efficiency [18]. Mettl3-mediated m^6^A modification plays a role in multiple biological events and complex diseases, such as spermatogenesis [19], stem cell pluripotency [20], postnatal liver development [21], and tumorigenesis [22]. Moreover, Mettl3 regulates lipid metabolism in the liver and participates in NAFLD progression [23–25]. However, the molecular mechanisms underlying Mettl3-mediated regulation of liver lipid metabolism and NAFLD progression have not been fully elucidated.

The present study aimed to investigate the roles of Mettl3 in liver lipid metabolism using Mettl3 hepatocyte-specific knockout (HKO) mice and multi-omics joint analysis of public data from the Gene Expression Omnibus (GEO) database. Our study revealed that Mettl3 deficiency in hepatocytes mediated m^6^A modification regulated lipid metabolism and non-alcoholic fatty liver disease (NAFLD) progression by significantly downregulating genes related to lipid metabolism. The newly identified lipid metabolism-related genes modified by Mettl3-mediated m^6^A modification may serve as potential targets for the treatment of NAFLD in the future.

## RESULTS

### Mettl3 expression is downregulated in liver tissues of patients and mice with obesity

**Figure 1:**
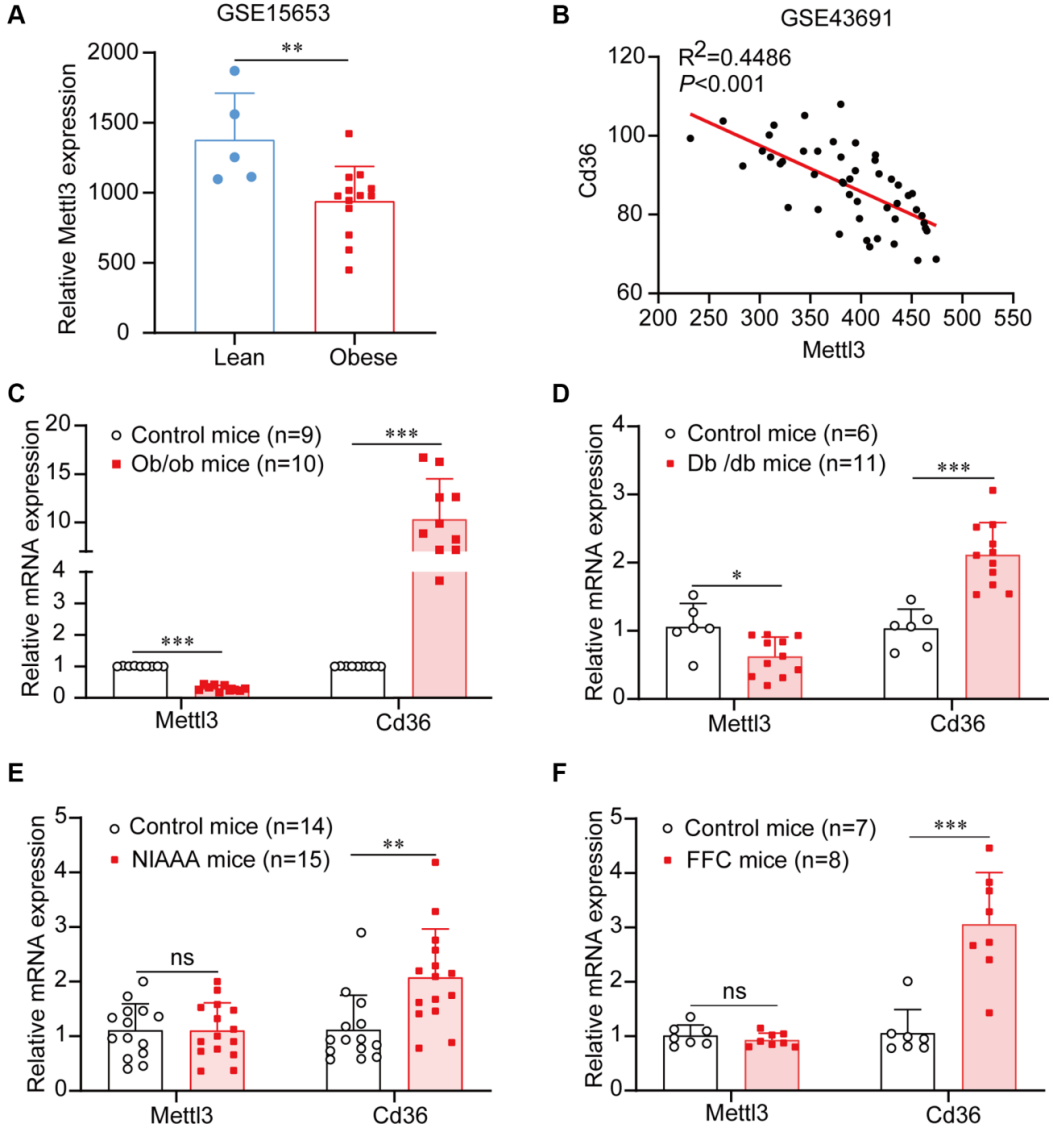
**Mettl3 expression is downregulated in liver tissues of patients and mice with obesity.** (**A**) Mettl3 expression is downregulated in the liver of patients with obesity (with or without type 2 diabetes) compared to that in lean individuals from the GEO dataset (GSE15653). (**B**) The expression of Mettl3 is significantly negatively correlated with Cd36 in liver of obese hyperphagic mice fed with the high-fat diet and control mice from the GEO dataset (GSE43691). (**C**–**F**) Relative expression of Mettl3 in the livers of ob/ob (**C**), db/db (**D**), NIAAA (**E**), and FFC (**F**) mice was detected by qRT-PCR. Data are presented as the mean ± SD. Abbreviation: ns: not significant; ^*^P < 0.05; ^**^P < 0.01; ^***^P < 0.001.

To determine the expression of Mettl3 in fatty liver, we first used a public gene expression dataset from the GEO database. We found that the mRNA expression of Mettl3 was significantly decreased in the liver of patients with obesity compared to that in lean individuals (Figure 1A). To further uncover the expression of Mettl3 with the progression of NAFLD, we analyzed the expression correlation of Mettl3 with Cd36 and Dgat1. As previously reported, the expression of Cd36 in the liver positively correlates with the progression of morbidly obese patients [26]. Dgat1 is a critical enzyme in triglyceride synthesis and NAFLD development [27–29]. Pearson’s correlation analysis revealed that the expression of Mettl3 in the liver was significantly negatively correlated with Cd36 and Dgat1 (Figure 1B and Supplementary Figure 1). Moreover, quantitative reverse transcriptase PCR (qRT-PCR) analysis revealed that the mRNA level of Cd36 was significantly upregulated in the liver of four different kinds of fatty liver disease mouse models (Figure 1C–1F). However, Mettl3 expression was significantly downregulated in the liver of diabetes (db/db) mice and obese (ob/ob) mice (Figure 1C, 1D) but not in the liver of alcohol abuse and alcoholism (NIAAA) mice and high saturated fat, cholesterol, and fructose (FFC) diets induced mice (Figure 1E, 1F) compared to the control. These results indicate that the downregulation of Mettl3 expression in the livers may be associated with specific fatty liver disease pathogenesis.

### Mettl3 HKO induces lipid accumulation in mouse hepatocytes

**Figure 2:**
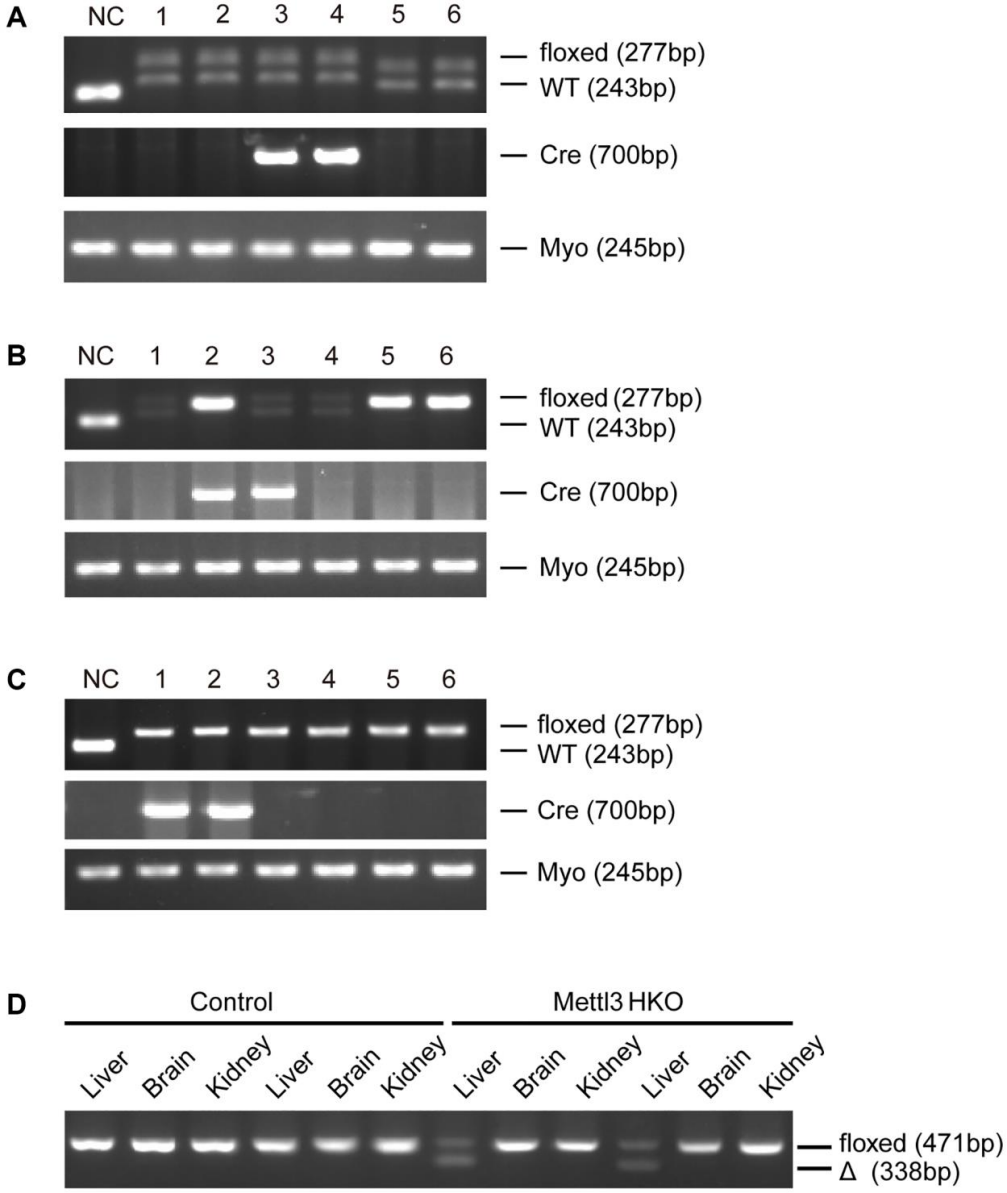
**Generation of hepatocyte-specific Mettl3 knockout mice.** (**A**) The offspring mice with different genotypes from intercrossing Mettl3^flox/flox^ and heterozygous Alb-Cre mice. (**B**) The offspring mice with different genotypes from intercrossing Mettl3^flox/wt^; Alb-Cre and Mettl3^flox/flox^ mice. (**C**) The offspring mice with different genotypes from intercrossing Mettl3^flox/flox^; Alb-Cre and Mettl3^flox/flox^ mice. (**D**) PCR-based genotyping of genomic DNA collected from the main organs of METTL3 HKO mice and control mice.

**Figure 3:**
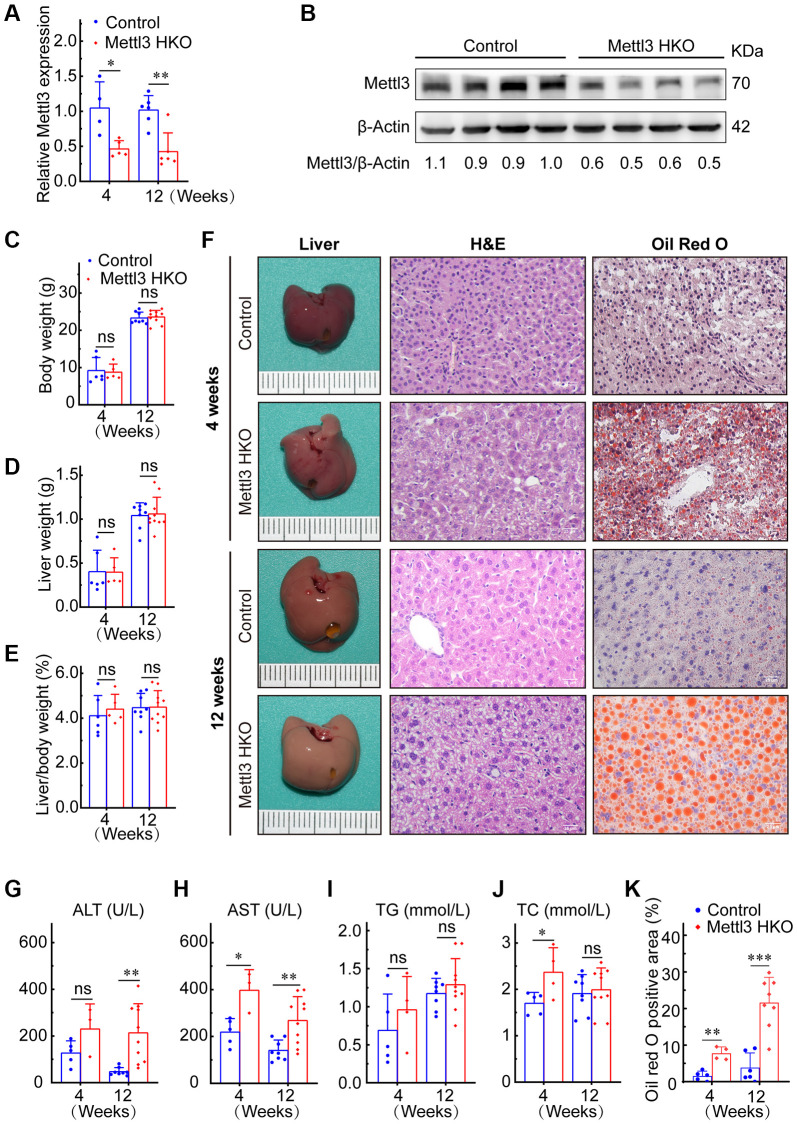
**Hepatocyte-specific deletion of Mettl3 induces lipid accumulation in mouse liver cells.** (**A**) qRT-PCR assay of the relative Mettl3 expression in control and METTL3 HKO mouse livers at 4 and 12 weeks. (**B**) Representative Western blot analysis and quantitative results in control and METTL3 HKO mouse livers. (**C**–**F**) Body weight (**C**), liver weight (**D**), and liver-to-body weight ratio (**E**) of METTL3 HKO and control mice at 4 and 12 weeks. (**F**) Representative images of the livers of METTL3 HKO (*n* = 5 for 4 weeks; *n* = 10 for 12 weeks) and control mice (*n* = 6 for 4 weeks; *n* = 8 for 12 weeks) stained with hematoxylin and eosin and Oil red O. Scale bars, 25 μm. (**G**–**K**) Serum levels of ALT (**G**), AST (**H**), TG (**I**), TC (**J**), and Oil red O positive area (**K**). Data are presented as mean ± SD. Abbreviations: TG: triglyceride; TC: total cholesterol; ns: not significant; ^*^P < 0.05; ^**^P < 0.01; ^***^P < 0.001.

To further determine the exact roles of Mettl3 in the progression of fatty liver disease, we crossed Mettl3^flox/flox^ mice with heterozygous Alb-Cre mice to obtain Mettl3^flox/wt^; Alb-Cre mice (Figure 2A). We further intercrossed Mettl3^flox/wt^; Alb-Cre mice with Mettl3^flox/flox^ mice to obtain Mettl3^flox/flox^; Alb-Cre mice (Figure 2B). The mice eventually used in the experiments were obtained by intercrossing Mettl3^flox/flox^; Alb-Cre with Mettl3^flox/flox^ mice (Figure 2C). The hepatocyte-specific knockout of Mettl3 was validated by agarose gel electrophoresis using liver, brain, and kidney tissues (Figure 2D). Moreover, qRT-PCR and Western blotting confirmed that the expression of Mettl3 was dramatically reduced in the liver of Mettl3 HKO mice (Figure 3A, 3B). To determine the function of Mettl3 in the liver, we dissected the livers of Mettl3 HKO and control mice at 4 and 12 weeks. The body weight (Figure 3C), liver weight (Figure 3D), and liver-to-body weight ratio (Figure 3E) were comparable between Mettl3 HKO and control mice at both 4 and 12 weeks. However, the mouse liver in the Mettl3 HKO group showed a more yellowish appearance than in the control group at 4 weeks. This difference became more pronounced at 12 weeks (Figure 3F). Consistent with this, H&E staining of mouse liver tissues at 12 weeks showed diffuse microsteatosis of hepatocytes (Figure 3F). Furthermore, Oil Red O staining indicated that large red lipid droplets were deposited in hepatocytes at both timepoints (Figure 3F, 3K). In conclusion, these results suggested lipid accumulation in the hepatocytes of METTL3 HKO mice.

### Mettl3 HKO induces lipid metabolism disorder and liver injury

Mettl3 HKO mice also showed higher serum alanine aminotransferase (ALT) activities at 4 and 12 weeks (Figure 3G). Serum aspartate transaminase (AST) activity was also significantly increased in Mettl3 HKO mice at both time points compared to that in control mice, and the difference was more pronounced at 12 weeks (Figure 3H). These results indicate that the hepatocyte deletion of Mettl3 results in progressive liver injury. We also observed a significant increase in serum total cholesterol (TC) levels in the METTL3 HKO group at 4 weeks (Figure 3J). Although the triglyceride (TG) level was slightly elevated in the METTL3 HKO group, it did not reach statistical significance at either time points (Figure 3I). Collectively, these results indicate that knockout of Mettl3 induced lipid dysmetabolism and caused liver injury in mice.

### Mettl3 HKO results in the downregulation of lipid metabolism-related gene expression

**Figure 4:**
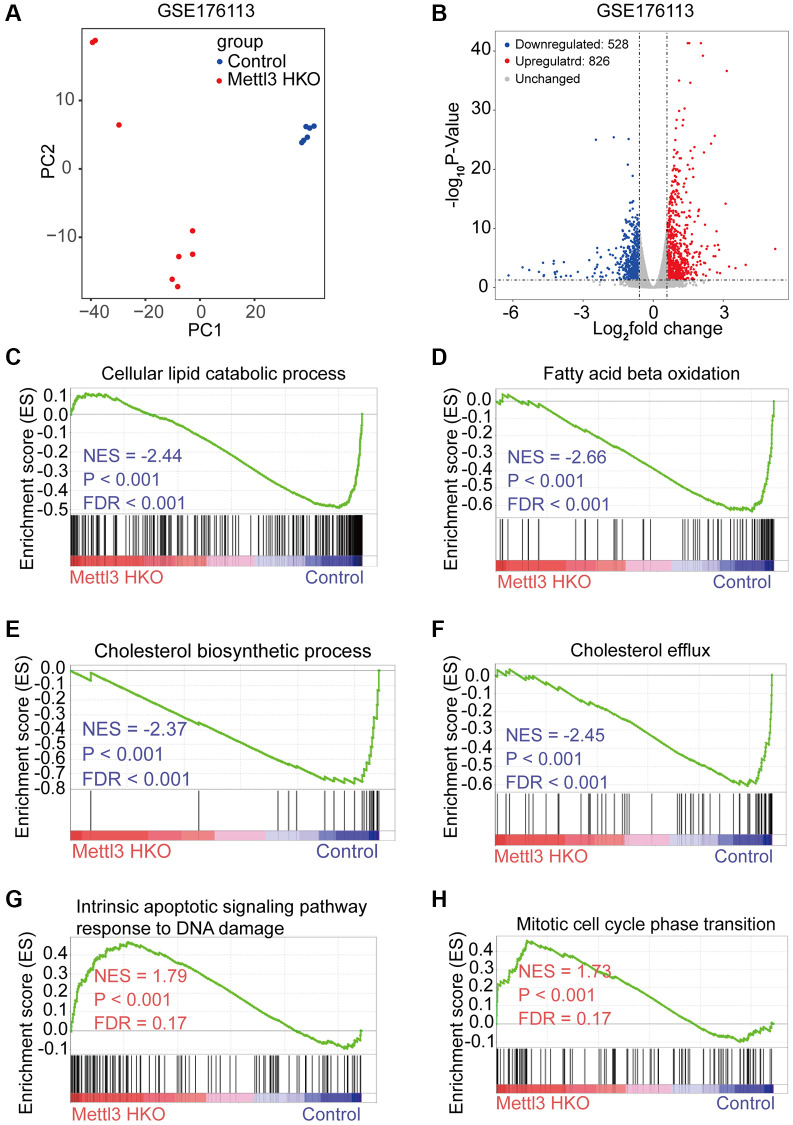
**Specific knockout of Mettl3 in hepatocytes results in downregulating lipid metabolism-related genes.** (**A**) Principal component analysis (PCA) plot showing reproducibility among the replicates of each group. (**B**) Volcano plot showing the differential expression of protein-coding genes in the GSE176113 dataset. (**C**–**H**) Gene set enrichment analysis (GSEA) plot of enrichment of indicated signatures in Mettl3 HKO livers using the C5 MSigDB database.

To reveal the molecular events underlying the effect of Mettl3 knockout on lipid metabolism, we analyzed a publicly available gene expression dataset of METTL3 HKO liver from the GEO database (GSE176113). PCA plot showed good reproducibility among the replicates of each group and apparent differences between the two groups (Figure 4A). Moreover, 826 protein-coding genes were upregulated, and 528 protein-coding genes downregulated significantly upon METTL3 HKO in this dataset (adjust-*P* < 0.05, fold change >1.5; Figure 4B). Gene set enrichment analysis (GSEA) using the curated gene set compilation M5 (MSigDb M5) demonstrated that several lipid metabolism and transport related-gene sets, including cellular lipid catabolic process, fatty acid beta-oxidation, cholesterol biosynthetic process, and cholesterol efflux, were significantly suppressed under the METTL3 HKO condition (|normalized enrichment score|>2.0; Figure 4C–4F), which may attribute to the phenotype of increased liver lipid accumulation and elevated serum cholesterol level in the METTL3 HKO mice. Moreover, several apoptosis and cell cycle-related gene sets were significantly positively enriched under the METTL3 HKO condition (|normalized enrichment score |>1.5; Figure 4G, 4H). These results indicate that METTL3 HKO caused lipid accumulation and subsequent apoptosis of hepatocytes, further leading to the compensatory proliferation of hepatocytes.

### Multiple omics analyses identify the specific targets of lipid disorder caused by Mettl3-mediated m^6^A modification

**Figure 5:**
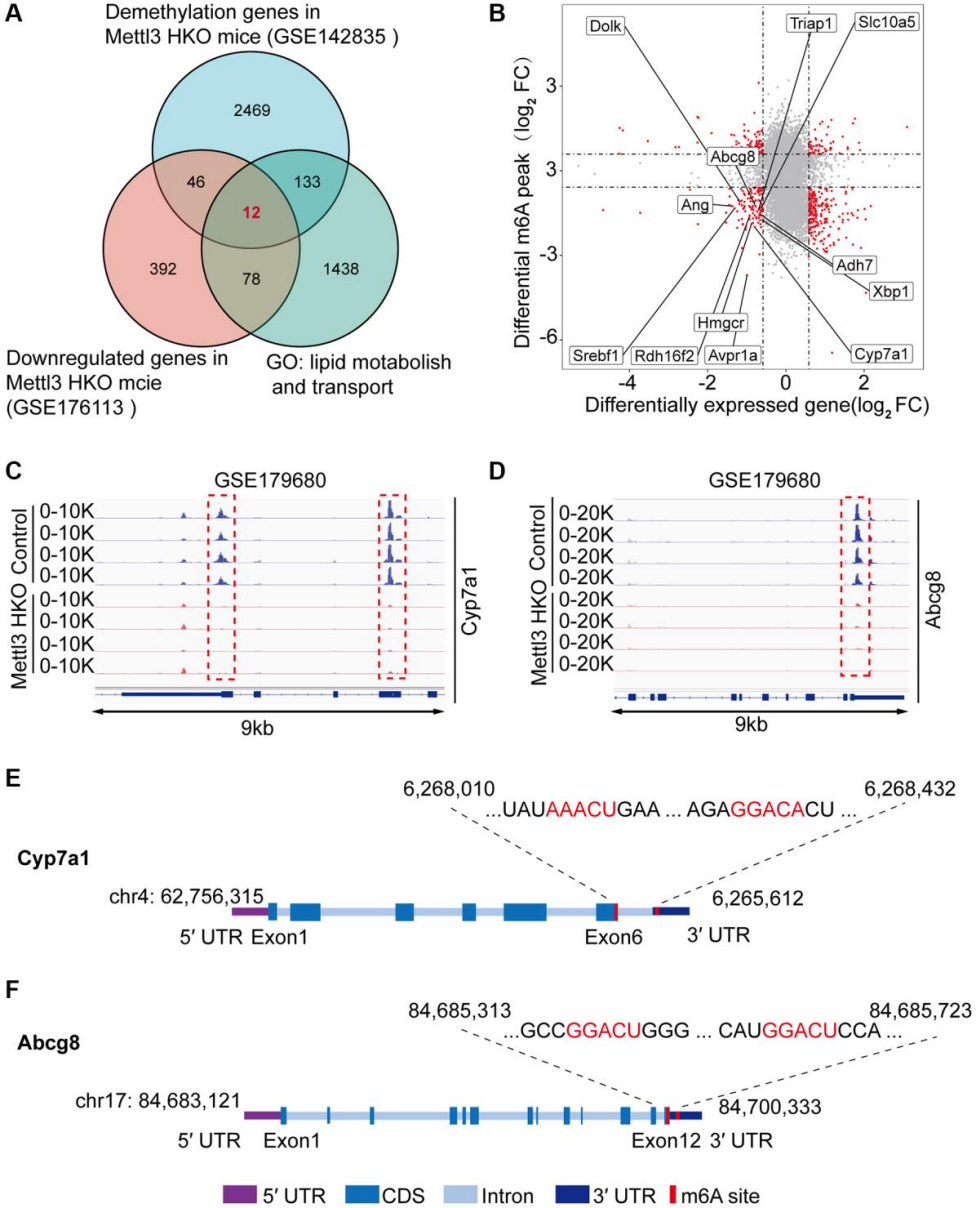
**Multiple omics analyses identify the specific targets of lipid disorder caused by Mettl3-mediated m^6^A modification.** (**A**) Venn diagram showing the number of overlapping Hypo-m^6^A-methylated mRNAs and downregulated genes in the liver of METTL3 HKO mice. Then the resultant 58 genes were annotated to GO term lipid metabolism and transport and obtained 12 genes. (**B**) Four-quadrant diagram showing the differentially methylated genes and differentially expressed genes in GSE142835 and GSE176113. (**C**, **D**) Peak distribution normalized to input in the genomic regions of Cyp7a1 (**C**) and Abcg8 (**D**) in control and METTL3 HKO mice in the GSE179680 dataset is shown. (**E**, **F**) Schematic representation of predicted positions of m^6^A motifs within Cyp7a1 (**E**) and Abcg8 (**F**) mRNA.

**Figure 6:**
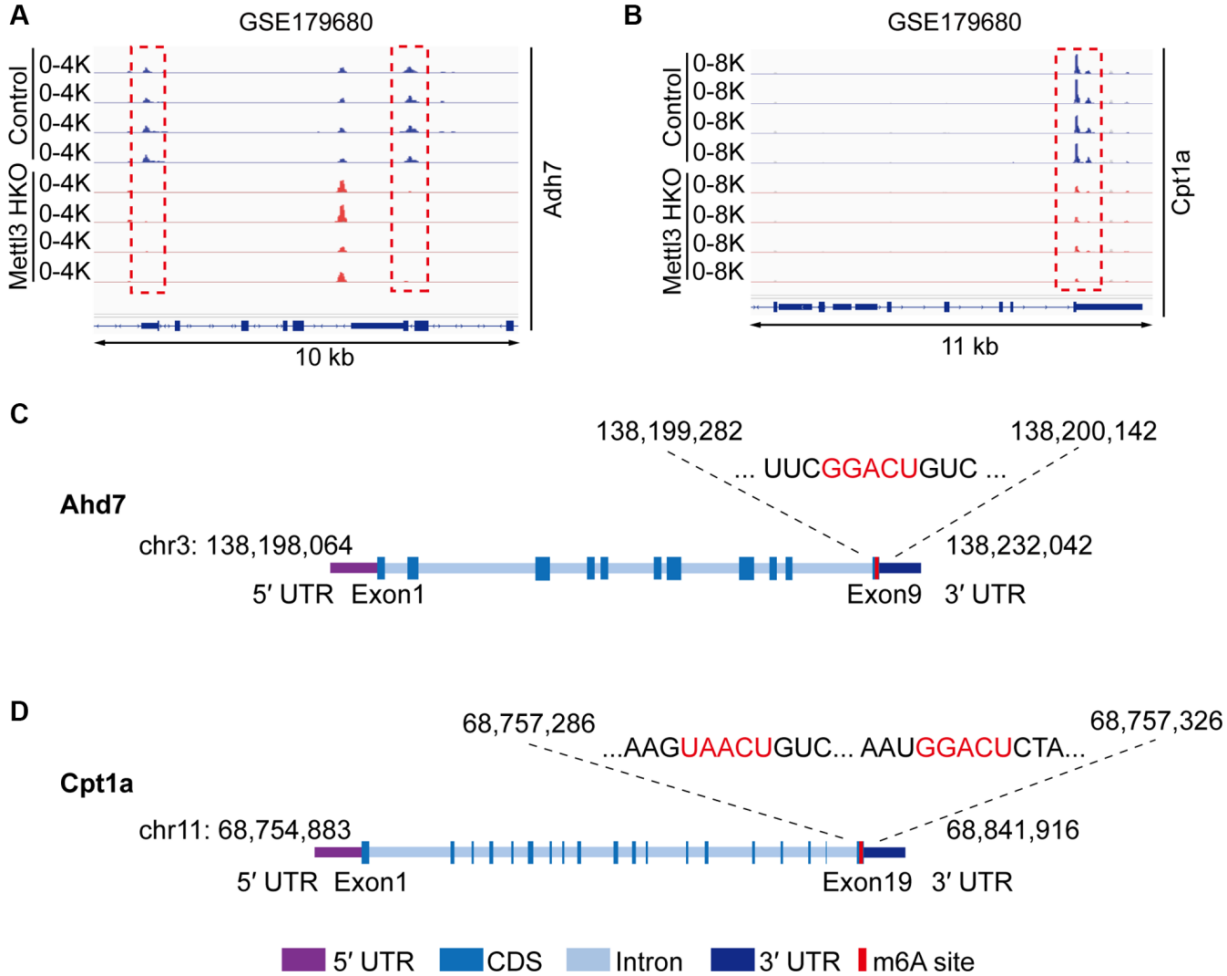
**Adh7 and Cpt1a are the potential targets modified by Mettl3-mediated m^6^A modification in regulating liver lipid metabolism.** (**A**, **B**) Peak distribution normalized to input in the genomic regions of Adh7 (**A**) and Cpt1a (**B**) in control and METTL3 HKO mice in the GSE179680 dataset. (**C**, **D**) Schematic representation of predicted positions of m^6^A motifs within Adh7 (**C**) and Cpt1a (**D**) mRNA.

To gain deeper insights into the molecular mechanism underlying the effects of Mettl3 HKO on liver lipid metabolism, we overlapped significantly demethylated genes (GSE142835) (|fold change|>2) with significantly downregulated genes under the Mettl3 HKO condition (GSE176113) (|fold change|>1.5) as well as genes related to lipid metabolism and transport. A total of 12 genes were identified (Figure 5A). The four-quadrant diagram showed that these genes were dramatically hypomethylated and downregulated (Figure 5B). The hypomethylation levels of these genes under the Mettl3 HKO condition were further validated by the GSE179680 dataset (data not shown). Cholesterol 7α-hydroxylase (Cyp7a1), which is involved in the catalysis of cholesterol synthesis of bile acids, and ATP-binding cassette subfamily G member 8 (Abcg8), which mediates hepatic cholesterol efflux, were dramatically hypomethylated in the Mettl3 HKO group (Figure 5C, 5D). In addition, most of these genes, including Cyp7a1 and Abcg8, had classical m^6^A “DRACH” motifs in the 3′UTR or near the stop codon (Figure 5E, 5F). Meanwhile, Ahd7 and Cpt1a, key enzymes in fatty acid oxidation [30, 31], were significantly demethylated in the liver of Mettl3 HKO mice (Figure 6A, 6B). Structurally, both Adh7 and Cpt1a have the classic “DRACH” sequence on the last exon (Figure 6C, 6D). These results indicate that Mettl3 may regulate lipid metabolism in hepatocytes by downregulating the expression of these lipid metabolism genes via m^6^A modification.

### Genes related to lipid metabolism are significantly downregulated in the liver of METTL3 HKO mice

**Figure 7:**
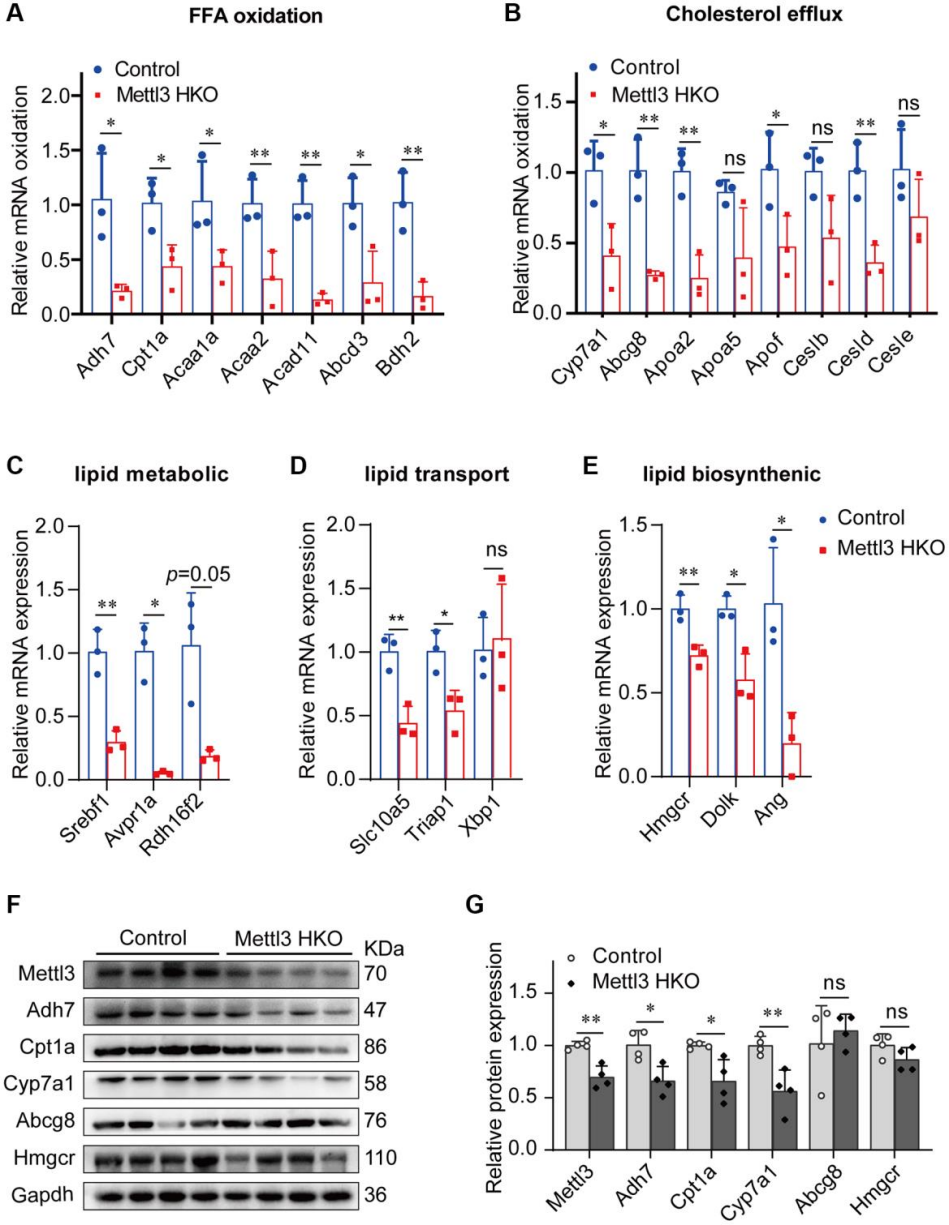
**Genes related to lipid metabolism are significantly downregulated in the liver of METTL3 HKO mice.** (**A**–**E**) qRT-PCR analysis of the mRNA expression of genes related to fatty acid oxidation (**A**), cholesterol efflux (**B**), lipid metabolic process (**C**), lipid transport (**D**), and lipid biosynthetic process (**E**) in the liver of METTL3 HKO mice and control mice. (**F**–**G**) Representative immunoblotting images (**F**) and quantitative results (**G**) for the potential target gene in the liver of METTL3 HKO mice and control mice. Data are presented as the mean ± SD. Abbreviations: FFA: free fatty acid; ns: not significant; ^*^P < 0.05; ^**^P < 0.01.

**Figure 8:**
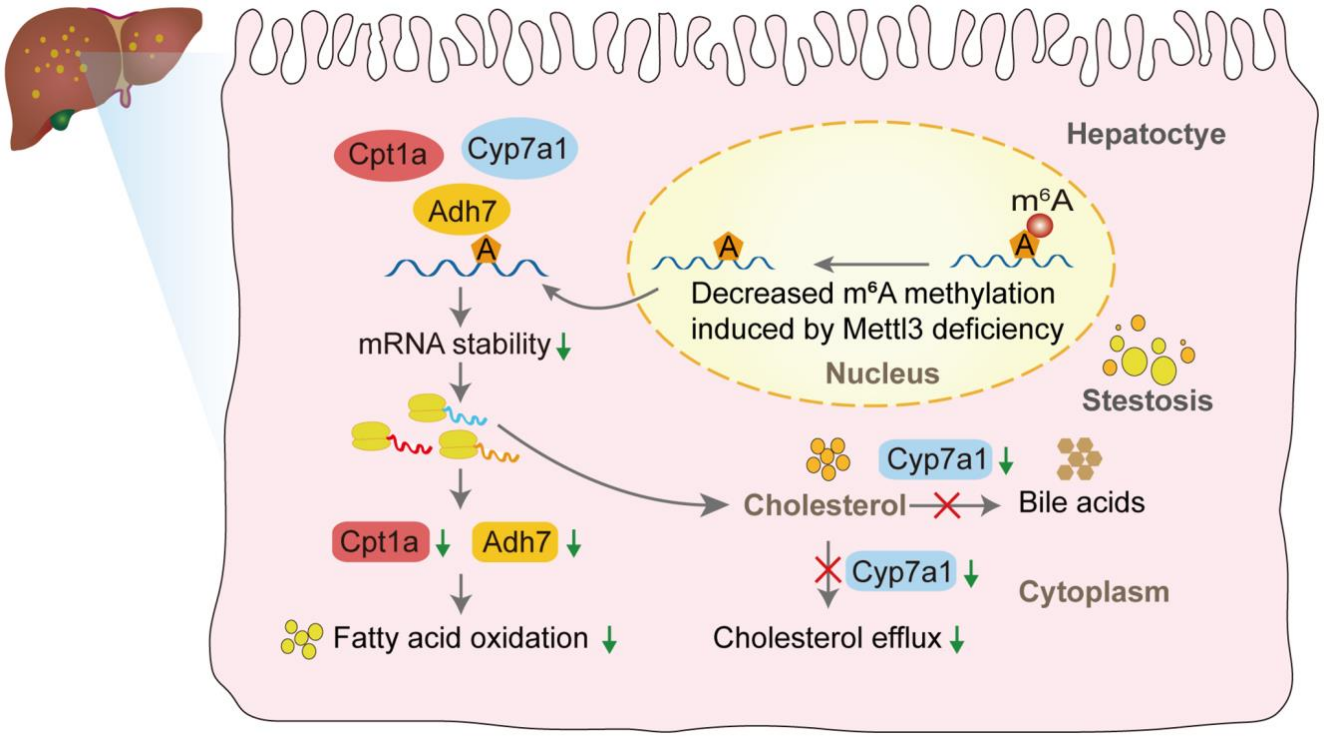
The proposed model of liver lipid accumulation through decreased m^6^A modification of the lipid metabolism genes mediated by Mettl3 deficiency.

We performed qRT-PCR to validate our hypothesis that Mettl3 regulates the expression of lipid metabolism-related genes via m^6^A modification. As expected, most of the genes related to fatty acid oxidation (Figure 7A), cholesterol efflux (Figure 7B), lipid metabolic process (Figure 7C), lipid transport (Figure 7D), and lipid biosynthetic process (Figure 7E) were significantly decreased in the livers of Mettl3 HKO mice. Moreover, we found an expression correlation of the potential target genes with Mettl3 (Supplementary Figure 2) using the GEO database. Furthermore, the protein expression of Adh7, Cpt1a, and Cyp7a1 was significantly downregulated in the liver of METTL3 HKO mice. While the expression of Abcg8 and Hmgcr remains unchanged between the two groups (Figure 7F, 7G). The significant downregulation of Adh7 and Cpt1a could further lead to decreased hepatocyte fatty acid oxidation, while the apparent downregulation of Cyp7a1 could decrease the conversion and transport of cholesterol in the liver. In conclusion, these results suggested that hepatocyte-specific Mettl3 deficiency-mediated significant downregulation of Adh7, Cpt1a, and Cyp7a1 may account for lipid accumulation and elevated serum cholesterol amounts in mice (Figure 8).

## DISCUSSION

Currently, NAFLD is a significant global health problem with no approved treatments. Dysfunction of liver lipid metabolism plays a vital role in the progression of NAFLD. However, the molecular mechanisms of liver lipid metabolism mediated by epigenetics have yet to be entirely understood. The present study found that decreased Mettl3 expression in NAFLD mouse models was associated with liver metabolic disorders. Using Mettl3 HKO mice, we observed significant lipid accumulation in the hepatocytes at 4 and 12 weeks. Moreover, increased serum levels of TC and progressive liver damage were observed in Mettl3 HKO mice compared to those in normal control mice at 4 and 12 weeks. Using public data from GEO for a multi-omics joint analysis and further molecular biology experiments, we identified potential targets modified by Mettl3-mediated m^6^A modification, such as Adh7, Cpt1a, and Cyp7a1, which regulate liver lipid metabolism. These results offer new insights into the molecular mechanism of m^6^A modifications in liver metabolism and may serve as a potential therapeutic target.

Previous studies have shown that the loss of Mettl3 in hepatocytes promotes lipid accumulation in the liver and contributes to the progression of NAFLD [21, 23, 24]. However, the exact molecular mechanism is not fully understood. The nuclear protein level of Mettl3 is significantly decreased in the liver of methionine-choline-deficient diet-induced nonalcoholic steatohepatitis (NASH) mice and patients with NASH but significantly increased in the liver of high-fat diet-induced NASH mice [23]. In our study, Mettl3 mRNA expression was significantly downregulated in the livers of db/db and ob/ob mice but kept unchanged in the livers of NIAAA and FFC mice. These results were seemingly contradictory. However, owing to the different etiology of fatty liver disease caused by different molecular mechanisms, Mettl3 may participate in the pathogenesis of fatty liver disease related to glucose metabolism disorders.

The present study found that hepatocyte Mettl3 knockout resulted in lipid accumulation in the liver, elevated serum TC levels, and progressive liver injury, consistent with a previous report [21]. The high amount of TG accumulation in the liver may be caused by the significant downregulation of genes involved in lipid catabolic processes and fatty acid oxidation, such as Adh7 and Cpt1a. As a critical ethanol-metabolizing enzyme, Adh7 participates in fatty acid omega-oxidation [31]. However, the roles of Adh7 in NAFLD and its epigenetic regulation by m^6^A modification have yet to be reported. Our study suggested that significant downregulation of Adh7 expression, regulated by Mettl3-mediated m^6^A modification, may contribute to NAFLD progression. Meanwhile, Cpt1a is a critical enzyme in fatty acid beta-oxidation, whose mRNA was also significantly demethylated around the stop codon in the liver of Mettl3 HKO mice. Moreover, the mRNA and protein expression of Cpt1a was also dramatically downregulated under the Mettl3 HKO condition. Therefore, this gene is likely to be regulated by Mettl3-mediated m^6^A modification and thus participate in forming the fatty liver caused by Mettl3 knockout. In addition, the significant increase in serum TC in our study may be caused by the combined effect of multiple genes related to cholesterol metabolism, including Cyp7a1, which plays a fundamental role in the regulation of hepatic cholesterol homeostasis by converting excess cholesterol to bile acids [32] and mediating cholesterol excretion via bile [33, 34]. In the present study, Cyp7a1 was significantly hypomethylated and downregulated, leading to decreased conversion and cholesterol transport into bile. These multiple effects eventually lead to a significant increase in serum cholesterol levels in Mettl3 HKO mice. Although a previous study has demonstrated the importance of cholesterol metabolism in NAFLD progression, few studies have reported the regulatory mechanism of cholesterol metabolism by m^6^A modification. Our study demonstrated, for the first time, that genes related to cholesterol metabolism, including Cyp7a1, may participate in NAFLD progression through Mettl3-mediated m^6^A modification.

In addition, there were nine other lipid metabolism-related genes, including Abcg8, Hmgcr, Srebf1, Triap1, Avpr1a, Dolk, Rdh16f2, Slc10a5, and Ang, which significantly lost their m^6^A modification and were downregulated in Mettl3 HKO mice. In these genes, Abcg8 worked as an essential enzyme mediating cholesterol excretion via bile [35]. Hmgcr is the enzyme catalyzing the rate-limiting step of cholesterol biosynthesis [36]. These genes were significantly downregulated at the mRNA level but kept unchanged at the protein level in METTL3 HKO mouse livers. Thus, their effect on NAFLD formation needs further study to illustrate. Srebf1 is a membrane-bound transcription factor that affects multiple biological processes of lipid homeostasis, including the synthesis of fatty acids, triglycerides, and cholesterol. Although the decreased expression of Srebf1 does not match the phenotype of hepatic lipid accumulation under the Mettl3 HKO condition, it may participate in hepatic lipid accumulation induced by Mettl3 loss, while its roles have been overshadowed by other genes. Except for Abcg8, Hmgcr, and Srebf1, the other genes’ roles in NAFLD need to be clarified. Thus, more profound studies are needed to clarify whether these genes contribute to the progression of NAFLD.

Our study has limitations. We did not validate Mettl3 protein expression in the liver of NAFLD mouse models and human NAFLD subjects. Moreover, the regulatory effect of m^6^A modification on mRNA fate is determined by a set of “reader” proteins after recognizing of m^6^A-modified transcripts, in which IGF2BP family proteins work to promote the stability of target mRNA [18]. Thus, we focused on genes significantly downregulated by Mettl3 HKO. However, if m^6^A modification is recognized by the “reader” protein YTHDF2 under Mettl3 HKO conditions, the expression of target genes is likely to be upregulated due to reduced degradation [37]. We also haven’t identified the “reader” protein under the Mettl3 HKO condition. Hence, future studies should address these issues.

## CONCLUSION

In summary, our study suggested the regulatory function of Mettl3-mediated epigenetic modification in mouse liver lipid metabolism, expanding our understanding of the regulatory network outside of transcriptomics in mammalian liver lipid metabolism and NAFLD pathogenesis. The newly identified lipid metabolism-related target genes may be potential targets for the treatment of NAFLD in the future.

## MATERIALS AND METHODS

### Animal experiments

All mice used in this study were raised in a specific pathogen-free-grade facility under a 12-h light/12-h dark cycle at 24 ± 2°C and humidity of 50% ± 10%. Mice were fed a standard chow diet with free access to water. All animal experiments were conducted in accordance with the National Institutes of Health Guide for the Care and Use of Laboratory Animals and were approved by the Animal Care and Use Committee of Southern Medical University.

### Generation of Mettl3^flox/flox^; Alb-Cre mice

Mettl3^flox/wt^ mice were generated using CRISPR-Cas9 system-assisted homologous recombination. Mettl3^flox/flox^ mice, in which exon 4 of Mettl3 was flanked by two loxp sites, were generated by mating Mettl3^flox/wt^ and Mettl3^flox/wt^ mice. The oligonucleotide sequences of the two sgRNAs were as follows: sgRNA1, GAAGTTACACTCTTTTAGGGAGG; sgRNA2, TAAACACCGGCCCTACGCCCAGG. Alb-Cre mice were purchased from Cyagen Biosciences (Suzhou, China). Mettl3^flox/flox^; Alb-Cre (Mettl3 HKO) mice were generated by crossing Mettl3^flox/flox^ mice with Alb-Cre mice. Both male and female mice were used for further experiments.

### Genotype analysis using PCR

Genomic DNA was prepared from mouse ear tissue using the TIANamp Genomic DNA Kit (Tiangen, Beijing, China), according to our previous publication [38]. Genotypes were determined by PCR using primers specific for Cre: 5′-ATCCGAAAAGAAAACGTTGA-3′ (forward), 5′-ATCCAGGTTACGGATATAGT-3′ (reverse); and specific for Mettl3: 5′-TAGTGCTGTGCCTTTCTTAG-3′ (Mettl3-L-LOXP-F), 5′-TTAAACTGACTGCCTCCATA-3′ (Mettl3-L-LOXP-R). The genomic DNA from a wild-type (WT) mice was used as a template. Moreover, to assess the knockout efficiency of Mettl3 in adult liver, the main organs and tissues DNA, including the liver was subjected to PCR using the following primer pair to amplify 338bp Mettl3 mutant fragment, F1: GTGCTGTGCCTTTCTTAG, R1: AGCGTCACTGGCTTTCAT, and R2: TTCTTGTTCTCCCCCAAT. Primer pairs for Myo were used as a negative control (forward primer: TTACGTCCATCGTGGACAGC, reverse primer: TGGGCTGGGTGTTAGCCTTA).

### Mouse models of fatty liver disease

All mouse models of fatty liver disease were generated using age-appropriate male mice with a C57BL/6 genetic background. Diabetes (db/db) mice and obese (ob/ob) mice were purchased from GemPharmatech Co., Ltd. (Jiangsu, China) (*n* = 11 for db/db mice, *n* = 10 for ob/ob mice). The National Institute on Alcohol Abuse and Alcoholism (NIAAA) model (*n* = 15) was induced by chronic and binge ethanol feeding as previously described [39]. The high saturated fat, cholesterol, and fructose (FFC) mouse model (*n* = 8) were induced by feeding with high fructose, high-fat, and high-cholesterol diet (research Dies: D09100310) for 24 weeks as previously reported [40].

### Histological analysis

Formalin-fixed, paraffin-embedded liver tissue samples were cut into 4 μm-thick sections and stained with hematoxylin and eosin (H&E) according to standard procedures. According to the manufacturer's instructions, liver lipid accumulation was confirmed using a Modified Oil Red O stain kit (Catalog No. C0158S; Beyotime, Beijing, China). Briefly, frozen liver slices (8 μm) were fixed in 10% formaldehyde for 10 min and then washed with 60% isopropanol for 20–30 s. Liver tissue was stained in Modified Oil Red O solution for 20 min. After staining, the slices were washed with 60% isopropanol and H_2_O.

### Serological test

The supernatant was obtained by centrifugation at 800 g for 10 min. The mouse serum levels of liver damage indices, blood lipids, and blood sugar were detected using the Beckman automatic biochemical analyzer AU680 (Beckman Coulter, Brea, CA, USA).

### RNA extraction and quantitative reverse transcriptase PCR

Total RNA was extracted from the mouse liver at the same anatomical position using TRIzol Reagent (TaKaRa) and reverse-transcribed into cDNA using the Reverse Transcription System (TaKaRa). qRT-PCR was performed using the SYBR Green qPCR Master Mix (TaKaRa) and a LightCycler 96 system (Roche). Relative gene expression was analyzed using the 2^ΔΔCt^ method with Gapdh as the internal control. All primers used in this study are listed in Supplementary Table 1.

### Western blotting

Total proteins from mouse liver tissues were extracted by Total Protein Extraction Kit (Keygen) and further quantified by BCA Protein Quantitation Kit (Keygen) according to the manufacturer’s instructions. Western blotting was performed as previously described with the following primary antibodies: anti-Mettl3 (1:1000; ab195352; Abcam), anti-β-actin (1:2000, 20536-1-AP; Proteintech Group), anti-Gapdh (1:5000, 10494-1-AP; Proteintech Group), anti-Adh7 (1:1000; 23425-1-AP; Proteintech Group), anti-Cpt1a (1:1000; 15184-1-AP; Proteintech Group), anti-Cyp7a1 (1:500; sc-293193; Santa Cruz), anti-Hmgcr (1:500; sc-271595; Santa Cruz), and anti-Abcg8 (1:1000; A01482-1; Boster).

### Public dataset

All the public datasets used in this study were obtained from the National Cancer for Biotechnology Information GEO. RNA transcriptome sequencing data were from patients with obesity (GSE15653) and Mettl3 HKO mice (GSE176113). The MeRIP-seq datasets GSE179680 and GSE142835 were from Mettl3-knockout livers. The results of principal component analysis (PCA), volcano map, four-quadrant diagram, and Venn diagram were visualized using the ggplot2 package [3.3.6]. GSEA was performed using GSEA software (version 4.3.2) downloaded from https://www.gsea-msigdb.org/gsea/index.jsp [41], using the curated gene set compilation M5 (MSigDb M5). The m^6^A modification peaks of target genes were visualized by Integrative Genomics Viewer (IGV) software (version 2.16.0) downloaded from https://igv.org/ [42]. The potential m^6^A sites (“DRACH,” where D = A, G or U; R = A or G; H = A, C or U) of the target genes were predicted using an online tool, SRAMP (http://www.cuilab.cn/sramp/) [43].

### Statistical analysis

Student’s *t*-test was used to compare two groups if the data met the normal distribution; otherwise, the Mann-Whitney U test was used. GraphPad Prism software (version 8.0) was used to analyze all data. Statistical significance was set at *P* < 0.05. Data are expressed as mean ± SD.

### Data availability

The data supporting this study’s findings are available from the corresponding author upon reasonable request.

## Supplementary Materials

Supplementary Figures

Supplementary Table 1
