# Supplementary material for: Mettl3-mediated m6A modification plays a role in lipid metabolism disorders and progressive liver damage in mice by regulating lipid metabolism-related gene expression
Source: Aging (Albany NY). 2023 Jun 16;15(12):5550–68. doi: 10.18632/aging.204810 (PMC10333091; doi:10.18632/aging.204810)
Supplement: Supplementary Figures [file aging-15-204810-s001.pdf]

SUPPLEMENTARY FIGURES

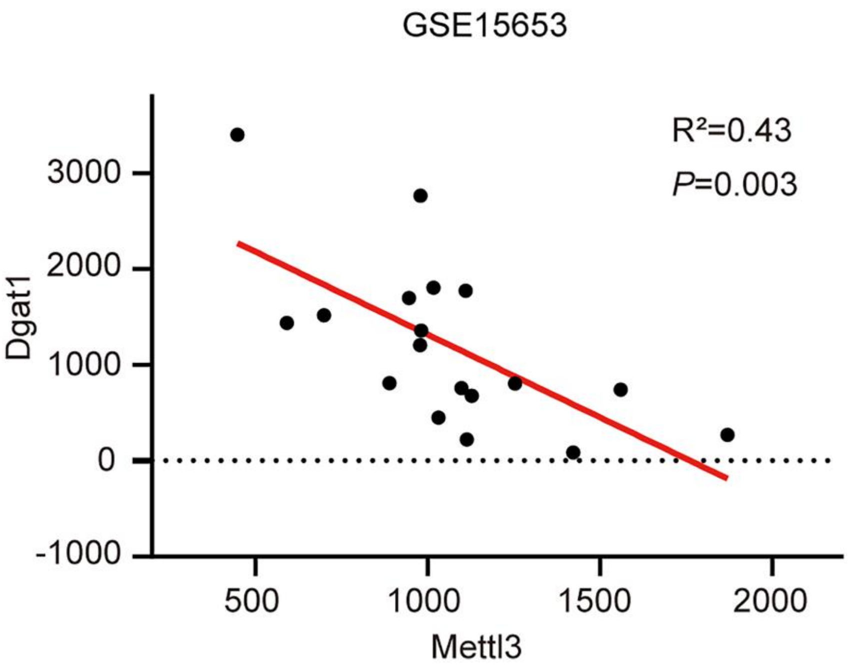

Supplementary Figure 1. The expression of Mettl3 is significantly negatively correlated with Dgat1 in GEO datasets.

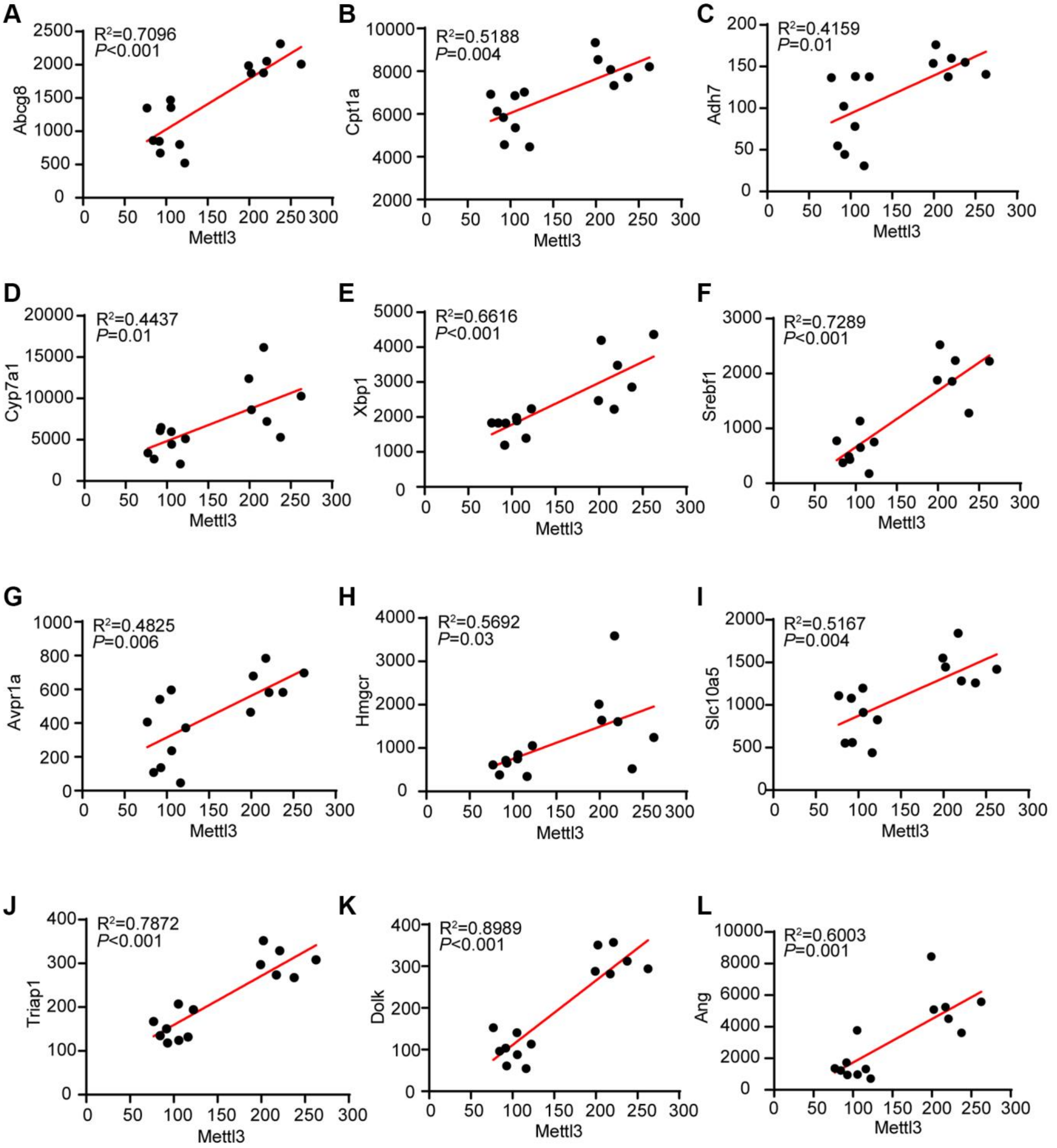

Supplementary Figure 2. The expression correlation of the potential downstream target genes with Mettl3. (**A**) The correlation between Abcg8, Cpt1a (**B**), Adh7 (**C**), Cyp7a1 (**D**), Xbp1 (**E**), Srebf1 (**F**), Avpr1a (**G**), Hmgcr (**H**), Slc10a5 (**I**), Triap1 (**J**), Dolk (**K**), Ang (**L**) expression and Mettl3 expression was determined by Pearson's correlation test from liver mRNA profiles of 5-week-old wild type and Mettl3 liver specific knockout mice in a GEO datasets (GSE176113).
