# Supplementary material for: Mettl3-mediated m6A modification plays a role in lipid metabolism disorders and progressive liver damage in mice by regulating lipid metabolism-related gene expression
Source: Aging (Albany NY). 2023 Jun 16;15(12):5550–68. doi: 10.18632/aging.204810 (PMC10333091; doi:10.18632/aging.204810)
Supplement: Supplementary Table 1 [file aging-15-204810-s002.pdf]

SUPPLEMENTARY TABLE

Supplementary Table 1. Primers for qRT-PCR analysis of lipid metabolism genes.

Gene	Forward primer (5′–3′)	Reverse primer (5′–3′)
Gapdh	CCTGCTTCACCACCTTCTTG	CATGGCCTTCCGTGTTCCTA
Mettl3	ATCCAGGCCCATAAGAAACAG	CTATCACTACGGAAGGTTGGG
Cd36	ATGGGCTGTGATCGGAACTG	GTCTTCCCAATAAGCATGTCTCC
Cyp7a1	GGGATTGCTGTGGTAGTGAGC	GGTATGGAATCAACCCGTTGTC
Abcg8	CTGTGGAATGGGACTGTACTTC	GTTGGACTGACCACTGTAGGT
Hmgcr	AGCTTGCCCGAATTGTATGTG	TCTGTTGTGAACCATGTGACTTC
Adh7	ATGGGCACCGCTGGAAAAG	TAACACGGACTTCCTTAGCCT
Cpt1a	CTCCGCCTGAGCCATGAAG	CACCAGTGATGATGCCATTCT
Srebf1	TGACCCGGCTATTCCGTGA	CTGGGCTGAGCAATACAGTTC
Avpr1a	TGAGTTTCGTTCTGAGCATACC	CCCAGCAATCTTGGGCTTTG
Rdh16f2	TCTTGGGCAGAGTGTCACTTG	TGCCAGGTATTTCTCTCCATAGA
Slc10a5	CAGCTACCTGCTCGTGAAGTT	AGGTTGACGGTAAAGTCTGTGA
Triap1	GAGTACGACCAGTGCTTCAAC	CTTGATTGCTTTCTGCACGCA
Xbp1	AGCAGCAAGTGGTGGATTTG	GAGTTTTCTCCCGTAAAAGCTGA
Dolk	CAGTGTGGGACCGATACTCCT	CCAAGCAAAGGCATGACCA
Ang	CCAGGCCCGTTGTTCTTGAT	GGAAGGGAGACTTGCTCATTC
Acaa1a	TCTCCAGGACGTGAGGCTAAA	CGCTCAGAAATTGGGCGATG
Acaa2	CTGCTACGAGGTGTGTTCATC	AGCTCTGCATGACATTGCCC
Acad11	TGACACCGTGGAAGTGCTAC	CCCGGCAAGTGCTGATTCA
Abcd3	GGCCTGCACGGTAAGAAAAGT	CCGCAATAAGTAACAAGTAGCCT
Bdh2	CGACTGGACGGCAAAGTTATT	CCTGGAGTTTGGACTCGTTGA
Apoa2	TGGTCGCACTGCTGGTAAC	TTTGCCATATTCAGTCATGCTCT
Apoa5	TCCTCGCAGTGTTCGCAAG	CGAAGCTGCCTTTCAGGTTCT
Apof	ATAGCCTCCGACTCATCCTGA	TCTGCATCTGGTATCCCAACTT
Ceslb	TACCTCCCCTGTTTTCCGAAG	GATGCTCCGCCTGTCATCAAT
Cesld	ATGCGCCTCTACCCTCTGATA	AGCAAATCTCAAGGAGCCAAG
Cesle	CAACTTCTGGAATTGATTGGGGA	GGGCTCCGGCATCTCTATG
